# Design and Optimization of Solid Lipid Nanoparticles Loaded with Triamcinolone Acetonide

**DOI:** 10.3390/molecules28155747

**Published:** 2023-07-29

**Authors:** Luigi Talarico, Simone Pepi, Surama Susino, Gemma Leone, Claudia Bonechi, Marco Consumi, Ilaria Clemente, Agnese Magnani

**Affiliations:** 1 Department of Biotechnology, Chemistry and Pharmacy, University of Siena, Via Aldo Moro 2, 53100 Siena, Italy; pepi11@student.unisi.it (S.P.); surama.susino@student.unisi.it (S.S.); gemma.leone@unisi.it (G.L.); claudia.bonechi@unisi.it (C.B.); marco.consumi@unisi.it (M.C.); ilaria.clemente2@unisi.it (I.C.); 2 National Interuniversity Consortium of Materials Science and Technology (INSTM)—Siena Research Unit, Via G. Giusti 9, 50121 Firenze, Italy; 3 Siena Research Group—Center for Colloids and Surface Science (CSGI), University of Florence, Via della Lastruccia 3, 50019 Firenze, Italy

**Keywords:** triamcinolone acetonide, solid lipid nanoparticles, drug delivery, experimental design, optimization

## Abstract

Principles of quality by design and design of experiments are acquiring more importance in the discovery and application of new drug carriers, such as solid lipid nanoparticles. In this work, an optimized synthesis of solid lipid nanoparticles loaded with Triamcinolone Acetonide is presented using an approach that involves Stearic Acid as a lipid, soy PC as an ionic surfactant, and Tween 80 as a nonionic surfactant. The constructed circumscribed Central Composite Design considers the lipid and nonionic surfactant quantities and the sonication amplitude in order to optimize particle size and Zeta potential, both measured by means of Dynamic Light Scattering, while the separation of unentrapped drug from the optimized Triamcinolone Acetonide-loaded solid lipid nanoparticles formulation is performed by Size Exclusion Chromatography and, subsequently, the encapsulation efficiency is determined by HPLC-DAD. The proposed optimized formulation—with the goal of maximizing Zeta potential and minimizing particle size—has shown good accordance with predicted values of Zeta potential and dimensions, as well as a high value of encapsulated Triamcinolone Acetonide. Experimental values obtained from the optimized synthesis reports a dimension of 683 ± 5 nm, which differs by 3% from the predicted value, and a Zeta potential of −38.0 ± 7.6 mV (12% difference from the predicted value).

## 1. Introduction

### Experimental Design Approaches on Lipid Nanocarriers

The principles of quality by design (QbD) are founded on a comprehensive and deep understanding of the manufacturing process and its influence on the final product. With the QbD process, the quality of the final product is not controlled by simply testing the final result but is implemented in the building process of the material, to further be optimized and modified according to the manufacturer’s needs [1,2]. The study of the main factors involved in a production process becomes relevant in nanoparticulate matter production, as small changes in the manufacturing process could result in very different final products. The actual unavailability of pharmaceutical products based on nanoparticulate systems is mainly explained by the manufacturer’s inability to control their quality and safety [3], as those systems are very susceptible to changes in their production processes. For this reason, is important to apply QbD-oriented approaches from early-stage research, as the identification of critical process parameters and critical material attributes could lead to a successful implementation of the desired product, with a robust production process that can be easily adjusted by identification of the principal factors that influence a certain final product’s property. Nanoparticulate products, in particular, require high standards of uniformity of samples in terms of dimensions and monodispersion of size populations [4]. In this context, the design of experiments (DoE) is the main statistical approach that leads to the identification of relevant parameters influencing the final product, using an approach that also allows investigating possible antagonism or synergy between the involved relevant factors [5].

**Figure 1:**
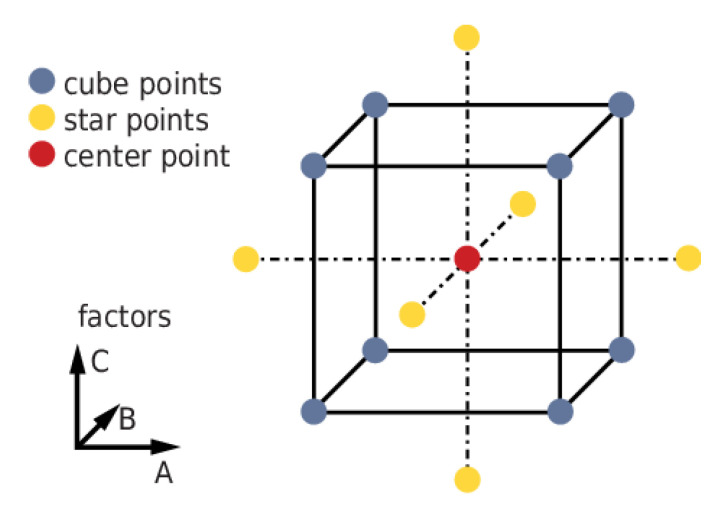
Scheme of a circumscribed Central Composite Design.

The identification of factors involved in a measured response is conducted by performing a variable number of experiments, which can be modified according to the desired resolution in the tested experimental space. The most basic approach to experimental design consists in performing a full factorial design, in which the number of factors studied is tested at two levels, usually codified as +1 (higher value) and −1 (lower value), for two-level designs, and −1, 0, and +1 for three-level designs [6,7]. This implies that the total number of runs to be performed varies as 2^n^ or 3^n^, and, for this reason, full factorial designs are usually implied for screening a minor number of variables. Other experimental designs, with reduced amounts of experimental runs, were created to overcome the exponential growth of samples to be analyzed. Plackett–Burman designs are considered effective screening designs of experiments when a large number of factors is involved, as they require only a number of experiments [8] that correspond to the first multiple of four higher than the factors to be screened. For this reason, these kinds of designs are also used as preliminary screening for more complete, full factorial designs. In the study performed by Shah et al., a Plackett–Burman design was implemented to study the influence of six parameters on the synthesis of Levofloxacin-loaded solid lipid nanoparticles (SLN), and then a three-level, full factorial design was used to monitor the most relevant factors selected and their influence on the responses with a higher degree of resolution [9]. Optimization designs, also known as surface response methodologies, are implemented on already consolidated processes or when previous screening on the relevant variables was performed. Each factor to be optimized is studied at least in three levels, allowing the study of quadratic and cubic terms to be included in the polynomial equation used to predict the behavior of a selected response, where a combination of independent factors could contribute in a synergistic (positive sign) or antagonistic way (negative sign) to the response [10]. The most-used kind of optimization design is the Central Composite Design (CCD), based on a classical full factorial design, with the addition of center points and the so-called *star points*, which are intermediate points located at a precise distance, called alpha, from the design center (Figure 1 [11]). The alpha value determines the kind of Central Composite Design obtained [5,12]. With the use of CCDs, the simultaneous optimization of parameters can be achieved through the use of desirability functions. In this way, a set of possible solutions is proposed in function of the established goal for each response, with an assigned desirability index (DI) from 0 to 1 that provides an overall measure of how well the combination of factors satisfies the desired goals.

Solid lipid nanoparticles are nanocarriers with a core-shell structure. The inner core is mainly composed of a lipid, solid at room and body temperature, while the protective shell consists of a single surfactant layer or a mixture of ionic and nonionic surfactants that stabilizes the nanoparticulate matter and reduces interfacial energy of those dispersion in water environments [13,14]. The main choices for the shell layer consist in a mixture of ionic and nonionic surfactants, with the most used being natural gums, polysaccharides such as chitosan [15], that could provide also a Zeta potential, or synthetical molecules, such as Tween, combined with different ionic surfactants. When used as stabilizer of nanoparticles, Tween has also shown reduced cytotoxicity [16] compared to its free form. SLNs have shown great encapsulation efficiency of lipophilic compounds [17,18], as well as the ability to modulate drug kinetics and improved physical stability during storage [19,20]. Among the lipids, fatty acids or esters with long saturated chains are usually preferred in the synthesis of solid lipid nanoparticles as they satisfy the high melting point requirement and for their greater mobility, which could lead to more imperfections in the crystal lattice, where lipophilic active ingredients are encapsulated. The actual localization of the payload depends on the active molecule’s nature. As solid lipid nanoparticles are able to encapsulate both hydrophobic and water-soluble compounds [21,22], three different models are proposed: a homogeneous distribution of drugs, a drug-enriched shell (for hydrophilic molecules), and a drug-enriched core for lipophilic compounds [23]. The transition of the crystalline lipidic phase to a lower energy state that consists of a more packed and ordered crystal lattice causes the release of active compounds from solid lipid nanoparticles [24,25], as well as precipitation and agglomeration of the nanoparticulate matter and the consequent progressive destruction of the system. SLNs are demonstrated to be versatile drug carriers for targeting various organs, and treat different diseases, such as cancer, pulmonary diseases, and ocular disfunctions and infections, targeting mainly the cornea and posterior eye segments [26]. Different examples of SLN delivering ocular drugs can be found in the literature, encapsulating natamycin, amphotericin B, and levofloxacin, also using design of experiments to optimize the synthetical process in terms of size, Zeta potential, and entrapment efficiency [27,28,29,30]. 

Many different synthetical routes can be used to synthetize solid lipid nanoparticles with different characteristics. Ultrasonic-assisted methods are among the most common routes, together with hot and cold homogenizations, double emulsions, solvent evaporation, and coacervation by precipitation of fatty acid salts [31,32]. Further functionalization of lipid nanoparticles can be implemented on the surface with other polymers to enhance biocompatibility, such as hyaluronic acid, polyethylene glycol, and chitosan, or single molecules such as folic acid [26,33,34]. More complex functionalization of the nanoparticles’ surfaces can also be achieved using antibodies [35].

SLNs are proposed as substitutes for conventional colloidal systems for their good biocompatibility and biodegradability and for their advantage over other lipidic nanoparticles of being synthesized without using organic solvents. Furthermore, the overall encapsulation efficiency of SLNs is higher compared to other lipidic nanocarriers capable of delivering lipophilic compounds, such as quercetin [18,36], and solid lipid nanoparticles are demonstrated to be resistant to freeze-drying and spray-drying processes, enhancing their shelf-life [37]. In the field of SLNs, Central Composite Designs have been widely used to optimize the physiochemical characteristics of those nanosystems, as they play a major role in the loading and stability of dispersions.

Triamcinolone Acetonide (TA) is an angiostatic corticosteroid that can be used against neovascularization in different environments, including eye segments [38,39,40], and it is also a lipophilic molecule with low solubility in water (21 µg/mL at 28 °C) [41]. Its low water solubility causes low bioavailability and permeation of biological membranes or mucosa [42], and delivery systems or permeation enhancer systems are usually required for subministration of Triamcinolone Acetonide.

Although the synthesis of solid lipid nanoparticles using Stearic Acid/Tween 80 is already reported and demonstrated to permeate membranes [43], fewer studies are focused on the optimization of the synthetical procedure and the exploration of experimental space with QbD approaches. Presented in this work is an optimized formulation of solid lipid nanoparticles loaded with Triamcinolone Acetonide (TA-SLN) prepared with hot oil in water emulsions exclusively assisted by ultrasonication and studied by means of quality by design and design of experiment (DoE) principles applying a Circumscribed Central Composite Design to key factors such as lipid quantity, surfactant quantity, and sonication power and exploring their influences on particle size and Zeta potential. The optimized formulation within the tested experimental space has the goal of minimizing dimensions and maximizing the Zeta potential of TA-SLN. The optimized formulation’s encapsulation efficiency is reported after purification via Size Exclusion Chromatography.

## 2. Results and Discussion

### 2.1. Central Composite Design

For each experimental run, the hydrodynamic diameter, expressed as Z-average (nm), and Zeta potential values were obtained. Table 1 lists the value obtained as mean ± standard deviation of three experiments, while size distributions graphs are reported in Figures S1–S20 of Supplementary Materials. Both models for particle size and Zeta potential were chosen on the basis of significance, concordance between adjusted and predicted R^2^, non-significance for the Lack of Fits, and ANOVA adequate precision, which ensures the signal-to-noise ratio of each model and guarantees its ability to distinguish significant measurements from statistical noise.

#### 2.1.1. Model Determination for Zeta Average

The rough dataset obtained for Zeta average was transformed to normalize data using an exponential equation (Equation (1)), as suggested by the Box–Cox equation (Lambda value −1.67).

(1) $$y^{\prime }=y^{-1.67}$$

The best model for particle size in relationship to the independent variables is found to be linear (overall model F-value 15.61, *p* = 0.0002, 95% CI, ANOVA adequate precision = 12.00). Table 2 reports the descriptive statistics of other models.

The variables included in the linear descriptive statistic were A, with an F-value of 19.67, *p* = 0.0005, 95% CI, B with an F-value of 21.17, *p* = 0.0006, 95% CI, and C with an F-value of 2.99 and *p* = 0.1092, 95% CI. Despite being not significant, the independent variable C was included in the final model because the elimination of this variable does not improve the model itself. The Lack-of-Fit Test for the linear model reports a non-significative fit for errors (F-value 1.07, *p*-value 0.6447, 95% CI) as proof of the goodness-of-fit between the data and the actual model. The final equation, expressed in terms of coded factors that predicts particle size in the tested experimental space, is reported in Equation (2).

(2) $${\left(\mathrm{Zeta}\;\mathrm{average}\right)}^{-1.67}=5.34\times 10^{-6}A-5.161\times 10^{-6}B+1.941\times 10^{-6}C$$

**Figure 2:**
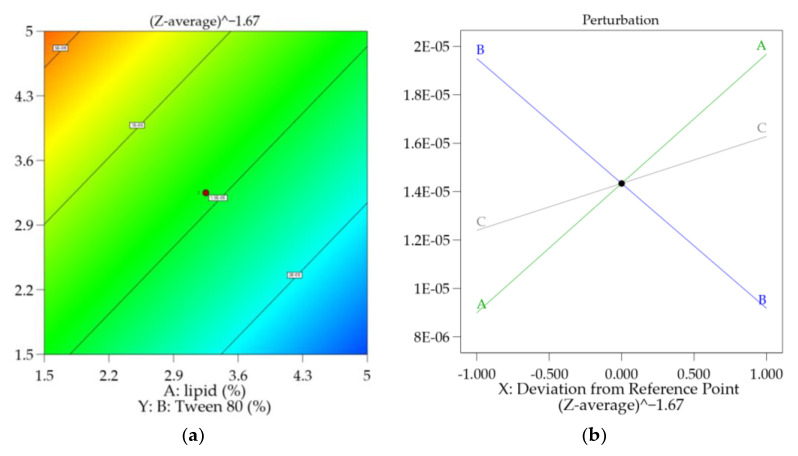
Contour (**a**) and perturbation plots (**b**) for the particle Zeta average model. Graphs are reported in the transformed scale. The red dot represents experimental design points.

Figure 2a shows the contour plot of Zeta average variation against the independent factors A and B, keeping the other variable C fixed at 52% of sonication power. The perturbation plot (Figure 2b) also shows the influence of C in the experimental space tested. The slope of each line represents the extent to which the Zeta average is influenced by changing each independent variable. It is noticeable how C (sonication power) has a minor influence on it, as it is more dependent on the concentration of lipid and anionic surfactant, while the sonication power has influence only on experiments where the concentration of reagents is fixed. As the model is linear, the effect obtained by changing one factor does not depend on the level of the others.

#### 2.1.2. Model Determination for Zeta Potential

The rough Zeta potential dataset was transformed to normalize the data using an inverse transformation (Lambda = −1, k = 49.5) (Equation (3))

(3) $$y^{\prime }=\frac{1}{y+49.5}$$

The best descriptive model for Zeta potential is found to be quadratic (overall model F-value = 26.35, *p* = 0.0007, 95% CI, ANOVA adequate precision = 17.15), as reported in Table 3.

Quadratic terms and two-factor combinations of A, B, and C are part of the descriptive model. To respect the hierarchy of combined variables, some factors are included in the model even with no statistical significance, as their exclusion has no effect on the overall model significance. The list of included variables, combined with their F-value and *p*-value, is reported in Table 4. The Lack-of-Fit test reports the highest non-significant *p*-value for the quadratic descriptive model, and the final equation expressed in terms of coded factors that predicts the behavior of Zeta potential among the tested experimental space is reported in Equation (4)

(4) $$\frac{1}{Zeta+49.5}=0.078-0.006A-0.031B-0.006C+0.0009AB+0.021AC\;-0.003BC-0.006A^{2}+0.033B^{2}$$

In a quadratic model that also contains terms which are the combination of independent variables, the result of changing the value of each one of the independent factors varies with respect of the level of the others in a synergistic or antagonistic way. In this case, the only antagonistic contribution is given by the term BC, as indicated by the negative sign in Equation (4).

**Figure 3:**
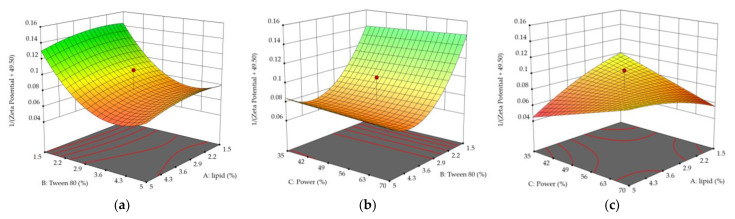
3D surface plots for AB (**a**), BC (**b**), and AC (**c**) terms. Each missing factor is fixed at its mean level. The red dot represents experimental design points.

Figure 3 reports the 3D plots of the AB (Lipid/Tween 80 Figure 3a), BC (Tween80/power, Figure 3b), and AC (Lipid/Power, Figure 3c) terms for Zeta potential. For each figure, the missing factor is fixed at its mean level. 

The main effects on the Zeta potential of solid lipid nanoparticles are given by the quantity of lipids and anionic surfactants, as those are the only reagents that could bring a net charge to the system. As the quantity of ionic surfactant is fixed among all the experimental runs, increasing the quantity of Tween 80 has always shown a lowering effect on the Zeta potential because of variation of the anionic/nonionic surfactant ratio. The percentage of non-ionic surfactant is the most influential factor on the Zeta potential of TA-SLN, as it is responsible for the major variations both against the lipid quantity (Figure 3a) and against sonication power (Figure 3b). Figure 3c shows the contribution to the Zeta potential of the lipid itself: Stearic Acid could contribute to the surface potential, as some of the carboxyl groups towards the nanoparticle’s surface could be present in the carboxylate form. Increasing lipid quantities and keeping the surfactant quantity fixed could lead to more negative SLN, even if the major contribution to Zeta potential is given to the ratio of the ionic/anionic surfactant.

### 2.2. Optimized Formulation

As the solutions to the experimental design proposed, an optimized formulation was elaborated assigning relative importance to the two different responses. Each proposed solution is identified by a desirability index (DI). Table 5 reports the optimal solution given where the minimization of the Zeta average is more important (++++) than the maximization of negative Zeta potential (+++). Relative importance was also chosen because all the formulations tested showed a Zeta potential higher than −25 mV, which is considered to be the prerequisite for electrostatic stability of suspensions. The corresponding desirability index for this solution is 0.81. Despite its high quantity, it was demonstrated by Gonçalves et al. that Tween 80 shows no cytotoxic effects under 3% [44].

**Figure 4:**
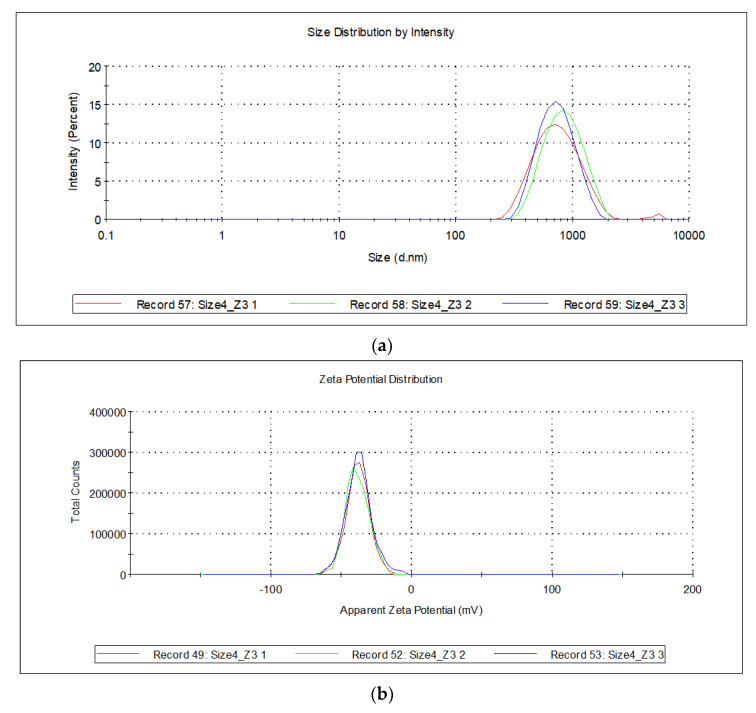
Experimental Size (**a**) and Zeta potential (**b**) distribution for the optimized formulation.

The experimental values for dimension and Zeta potential analysis for a sample prepared with the proposed quantities of lipid, non-ionic surfactant, and sonication power report a Zeta average corresponding to 683.4 ± 4.6 nm (PDI 0.24 ± 0.02) and a Zeta potential of −38.0 ± 0.6 mV. The obtained values are 3% higher than the predicted value for particle size and 12.4% higher than predicted for Zeta potential. Figure 4a,b reports the size distribution by intensity (Figure 4a) and Zeta potential distribution (Figure 4b) of the optimized formulation.

### 2.3. Encapsulation Efficiency of the Optimized Formulation

The encapsulation efficiency of the optimized TA-SLN formulation is calculated as previously reported, interpolating the peak area of TA obtained from the purification of a TA-SLN sample with a calibration curve (R^2^ = 0.9992 and equation y = 145122x) (LOD 0.1 ppm, LOQ 1 ppm). Using Size Exclusion Chromatography as a purification method, the resulting encapsulation efficiency of the TA-loaded solid lipid nanoparticles is equal to 94 ± 4%.

## 3. Materials and Methods

### 3.1. Materials

Absolute ethanol, Stearic Acid (≥98%), Tween 80 (for synthesis), L-α-phosphatidylcholine (from soybean, type II-S, 14–29% choline basis), Triamcinolone Acetonide (Analytical Standard), Acetonitrile (hypergrade, for LC-MS), formic acid (98–100% for HPLC LiChropur™) and Sephadex^®^ G-50 were purchased by Sigma Aldrich (Milano, Italy).

### 3.2. Preparation of TA-SLN

Solid lipid nanoparticles were prepared by an ultrasonic-assisted hot oil in water emulsion method [45,46]. The lipid phase composed of Stearic Acid and soy PC was melted under magnetic stirring and kept 10 °C above the Stearic Acid melting point, which is 69.3 °C. A volume of 500 µL of a standard solution of Triamcinolone Acetonide (1 mg/mL in ethanol) was added in the lipid phase. Separately, the aqueous phase containing the non-ionic surfactant (Tween 80) was brought to the same temperature and slowly added to the melted lipid phase. The O/W mixture was kept under magnetic stirring (400 rpm) and at 79 °C for 30 min to create a pre-emulsion and to let the ethanol evaporate, and it was then sonicated in an ice bath for 10 min, with 50% on/off cycles using a probe sonicator (Bandelin Sonoplus HD2070 equipped with a UW 2070 probe, BANDELIN electronic, Berlin, Germany). The resulting solid lipid nanoparticle dispersions were stored at 4 °C until further characterization. All of the tests on the prepared formulation were measured on the same day as the synthesis.

### 3.3. Particle Size and Zeta Potential Measurements

For each of the experimental runs, Dynamic Light Scattering was used to determine the hydrodynamic diameter and Zeta potential of the synthesized systems. Measurements were performed with a Malvern Zetasizer ZS90, (Malvern, Worcestershire, UK) on a diluted sample (5% *v/v*) at 25 °C and a 90° detector angle. All of the values reported for both Zeta average and Zeta potential are the mean of three experiments.

### 3.4. Experimental Design

The statistical analysis for the optimization was conducted with Design Expert 13, (StatEase, Minneapolis, MN, USA) with a circumscribed Central Composite Design (alpha = 1.68). The independent optimization factors are listed in Table 6, where the *w/v* percentage refers to the total volume of the final dispersion (10 mL).

Monitored responses were Zeta average as the index of dimensions and Zeta potential. Table 7 reports the suggested synthetical runs proposed by the Central Composite Design.

### 3.5. Purification from Unentrapped Triamcinolone Acetonide

Size Exclusion Chromatography was used to separate the unentrapped Triamcinolone Acetonide from the TA-SLN. A volume of 1 mL of TA-SLN was deposited on a packed column (30 × 2.5 cm) of swollen-up Sephadex^®^ G-50, (Cytiva, Marlborough, MA, USA). Fractions corresponding to 3 column volumes were collected, and the ones containing Triamcinolone Acetonide were combined and concentrated to determine the overall encapsulation efficiency (EE%) of TA-SLN.

### 3.6. HPLC-DAD Method for Detection and Quantitation of Triamcinolone Acetonide

Quantification of Triamcinolone Acetonide was achieved using a Thermo Fisher UltiMate 3000 HPLC-DAD setup, equipped with a Kinetex C18 Polar column (250 × 2.1 mm, 100 Å porosity, 2.6 µm particle size, Phenomenex, Torrance, CA, USA). The column oven was kept at 40 °C during all the experiments, using an isocratic method with A: H_2_O 0.1% *v/v* formic acid and B: Acetonitrile 0.1% *v/v* formic acid at the ratio of A:B = 60:40 (% *v/v*). Chromatograms were recorded at 240 nm. The sample injection volume was fixed at 3.00 µL. For the quantitation of Triamcinolone Acetonide from purified SLN samples, a linear calibration curve was obtained between the concentration ranges of 0.5 and 25 ppm. The encapsulation efficiency of the optimized formulation of TA-SLN was obtained using Equation (5):

$$EE(\%)=\frac{TA_{total}-TA_{unentrapped}}{TA_{total}}\times 100$$

## 4. Conclusions

This work reports the optimization of the synthetical process for Triamcinolone Acetonide-loaded solid lipid nanoparticles using Stearic Acid as a lipid, and Tween 80 and soy PC as nonionic and ionic surfactants, respectively, in compliance with quality by design principles. The Central Composite experimental design implemented has shown a linear dependence between the tested independent variables and Zeta average, with the major influence given by lipid and surfactant quantities, while the relationship of independent variables with Zeta potential follows a quadratic model. The Zeta potential is strongly influenced by the quantity of nonionic surfactants in relationship to lipid quantity and sonication power, due to different ratios between anionic and non-ionic surfactants in the formulation, while the minimum positive contribution is given by the AC term (combination of lipid quantity and sonication power) due to the possible presence of some Stearic Acid in the form of stearate anions on the particle surface. From the construction of response surfaces, an optimized synthetical pathway is obtained using 5% (*w/v*) of Stearic Acid, 2.45% (*w/v*) of Tween 80, and the fixed sonication power of 35%. This solution has the highest desirability index when assigning more relative importance to reduction of Zeta average with respect to maximizing of Zeta potential. Experimental values of Zeta average and Zeta potential are replicable and in good accordance with predicted results. When the solid lipid nanoparticles are purified via Size Exclusion Chromatography, this optimized synthesis has also shown a very high encapsulation efficiency, demonstrating that the proposed synthesis of solid lipid nanoparticles is capable of encapsulating Triamcinolone Acetonide in high quantities, yet permeation studies for the optimized formulation are required to classify this optimized formulation as drug delivery systems.

## Figures and Tables

**Table 1:** Zeta average, polydispersity index, and Zeta potential values obtained for each experimental run.

Run	Z-Average (nm)	PDI	Zeta Potential (mV)
1	733 ± 44	0.16 ±0.14	−39.5 ± 0.7
2	628 ± 4	0.43 ± 0.05	−43.3 ± 1.3
3	843 ± 60	0.18 ± 0.09	−43.6 ± 0.6
4	2418 ± 232	0.21 ± 0.12	−38.9 ± 2.3
5	869 ± 62	0.26 ± 0.30	−39.8 ± 0.1
6	917 ± 64	0.22 ± 0.07	−39.7 ± 0.1
7	735 ± 10	0.16 ± 0.15	−41.3 ± 0.5
8	933 ± 63	0.30 ± 0.11	−39.9± 0.2
9	833 ± 60	0.27 ± 0.05	−39.8 ± 0.2
10	656 ± 71	0.34 ± 0.30	−31.1 ± 0.7
11	2986 ± 712	0.44 ± 0.15	−32.1 ± 1.7
12	867 ± 61	0.25 ± 0.15	−39.7 ± 0.1
13	1824 ± 60	0.59 ± 0.11	−35.5 ± 1.8
14	550 ± 3	0.35 ± 0.08	−45.0 ± 1.9
15	711 ± 40	0.24 ± 0.09	−29.5 ± 0.9
16	693 ± 13	0.27 ± 0.01	−33.7 ± 0.9
17	590 ± 8	0.26 ± 0.05	−35.6 ± 1.3
18	751 ± 16	0.17 ± 0.05	−37 ± 1
19	637 ± 26	0.19 ± 0.09	−36.8 ± 0.4
20	2274 ± 188	0.31 ± 0.20	−41.8 ± 1.3

**Table 2:** Descriptive statistics of tested models on Zeta average.

Model	*p*-Value	Lack of Fit *p*-Value	F-Value	Adj R^2^	Predicted R^2^
Linear	0.0002	0.6447	15.61	0.7451	0.6239
Two-Factor Interaction	0.2889	0.6646	1.46	0.7715	0.5280
Quadratic	0.4568	0.6479	0.99	0.7711	0.3553

**Table 3:** Descriptive statistics of tested models in Zeta potential.

Model	*p*-Value	Lack of Fit *p*-Value	F-Value	Adj R^2^	Predicted R^2^
Linear	0.0990	0.4731	2.62	0.2445	−0.1097
Two-Factor Interaction	0.5917	0.4468	0.66	0.1765	0.0385
Quadratic	0.0007	0.9710	26.35	0.9129	0.8068

**Table 4:** Factors included in the predictive model for Zeta potential (CI 95%).

Factor	F-Value	*p*-Value
A: Lipid	2.63	0.15
B: Tween 80	71.56	<0.0001
C: Power	0.02	0.87
AB	0.03	0.85
AC	20.34	0.002
BC	0.31	0.59
A^2^	2.46	0.16
B^2^	64.04	<0.0001

**Table 5:** Values of independent variables and predicted variables for the optimized solution.

**Independent Variables**	**Solution Value**	
A: Lipid (% *w/v*)	5	
B: Tween 80 (% *w/v*)	2.45	
C: Power (%)	35	
**Predicted Variables**	**Goal**	**Value**
Zeta average (nm)	Minimize	663.2
Zeta potential (mV)	Maximize	−33.8
Desirability index		0.81

**Table 6:** Independent variables included in the experimental design.

Independent Variables	Levels	
	−1	+1
A: Lipid (% *w/v*)	1.5	5.0
B: Tween 80 (% *w/v*)	−1.5	5.0
C: Sonication Power (%)	35	70

**Table 7:** Experimental Runs included in the CCD DoE.

Run	A: Lipid (% *w/v*)	B: Tween 80 (% *w/v*)	C: Power (%)
1	5.00	1.5	35
2	5.00	1.5	70
3	1.50	1.5	35
4	1.50	5.00	35
5	3.25	3.25	52
6	3.25	3.25	52
7	1.50	1.50	70
8	3.25	3.25	52
9	3.25	3.25	52
10	6.20	3.25	52
11	1.50	5.00	70
12	3.25	3.25	52
13	0.30	3.25	52
14	3.25	0.30	52
15	5.00	5.00	35
16	3.25	3.25	52
17	3.25	3.25	82
18	3.25	3.25	23
19	5.00	5.00	70
20	3.25	6.20	52

## Data Availability

Data sharing not applicable.

## Supplementary Materials

The following supporting information can be downloaded at: https://www.mdpi.com/article/10.3390/molecules28155747/s1. Size distribution graphs are available online: Figures S1–S20 contains size distribution by intensity for each experimental run.
