# Supplementary material for: Design and Optimization of Solid Lipid Nanoparticles Loaded with Triamcinolone Acetonide
Source: Molecules. 2023 Jul 29;28(15):5747. doi: 10.3390/molecules28155747 (PMC10420805; doi:10.3390/molecules28155747)
Supplement: Supplementary file 1 [file molecules-28-05747-s001.zip › molecules-2491945-supplementary.pdf]

Talarico, L.; Pepi, S.; Susino, S.; Leone, G.; Bonechi, C.; Consumi, M.; Clemente, I.; Magnani, A.

Design and optimization of Solid Lipid Nanoparticles loaded with Triamcinolone Acetonide

SUPPORTING INFORMATIONS

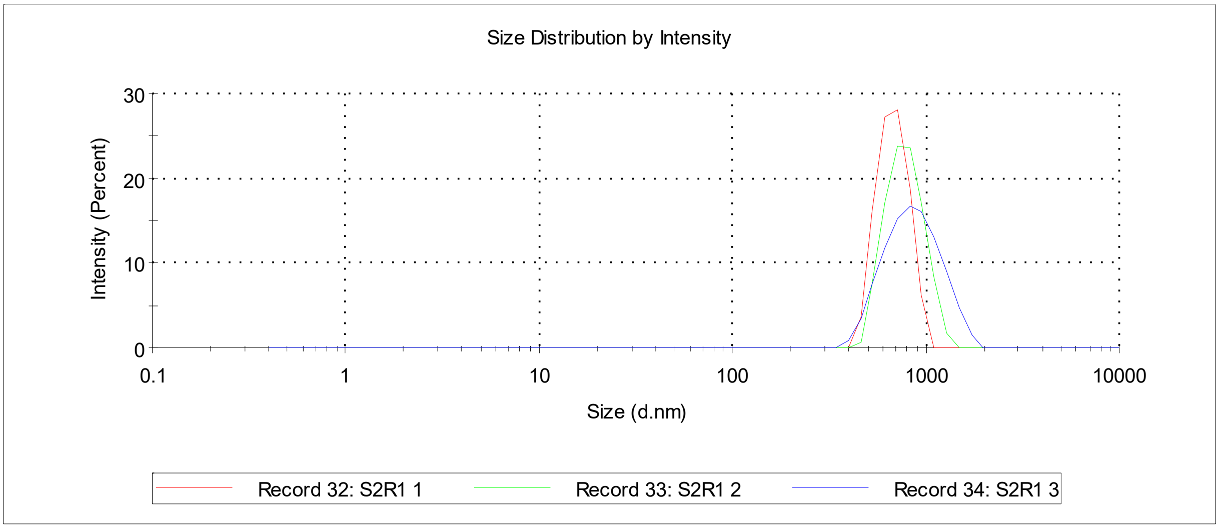

Figure S1: Size distribution of run 1

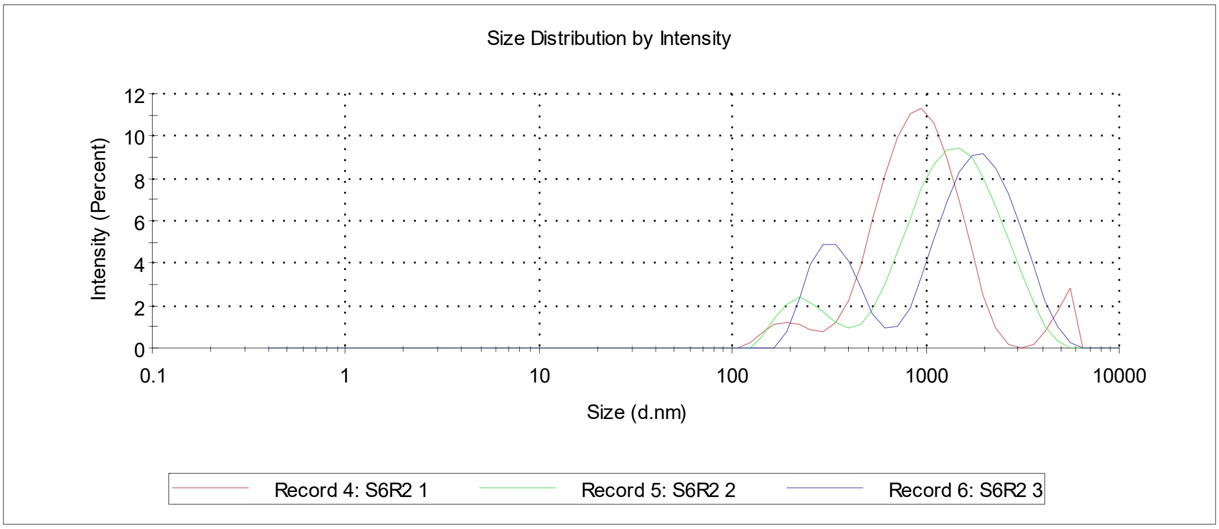

Figure S2: Size distribution of run 2

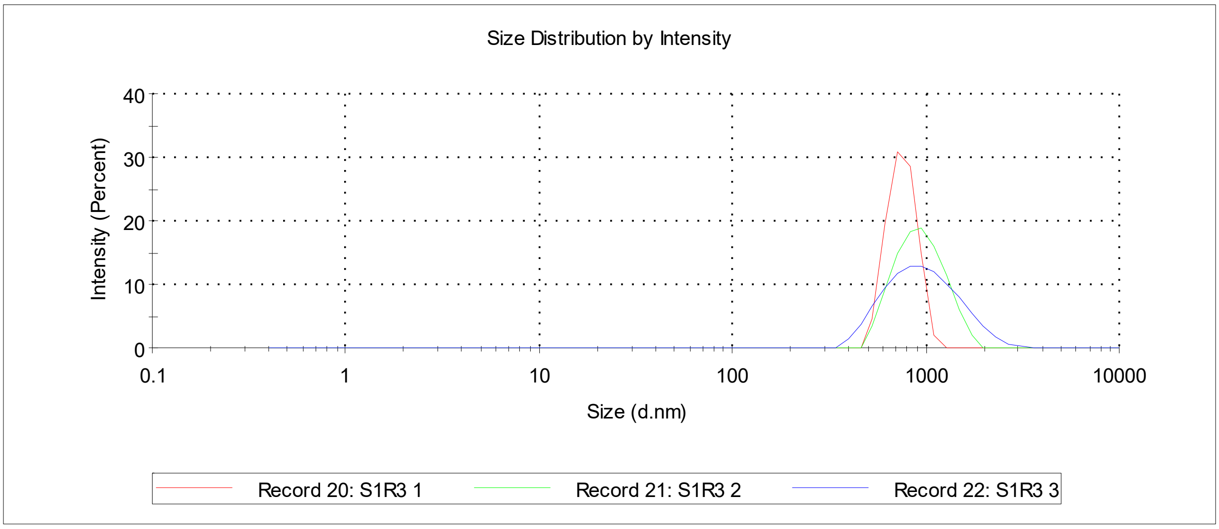

Figure S3: Size distribution of run 3

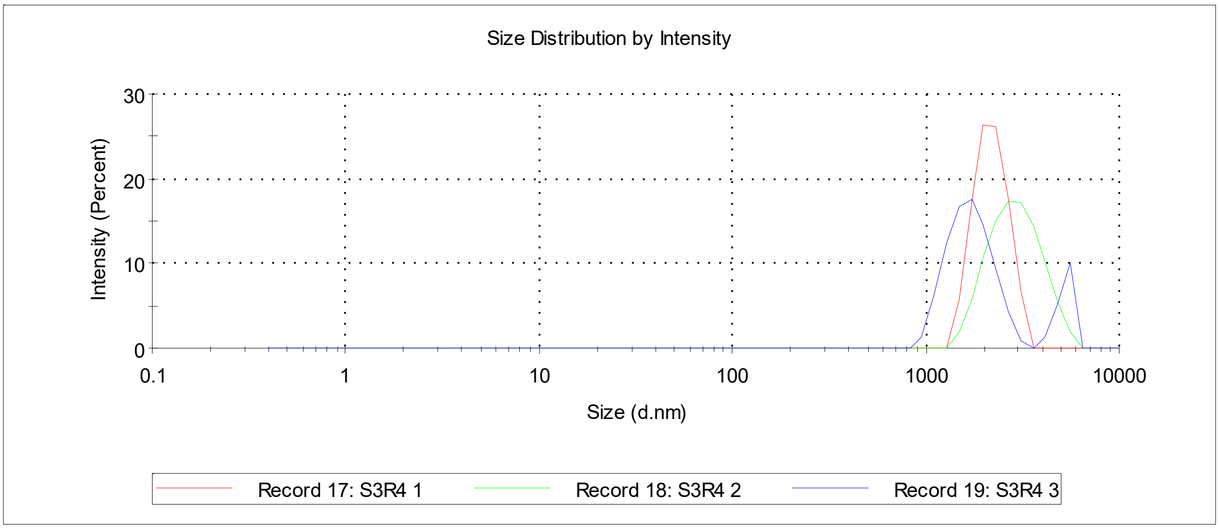

Figure S4: Size distribution of run 4

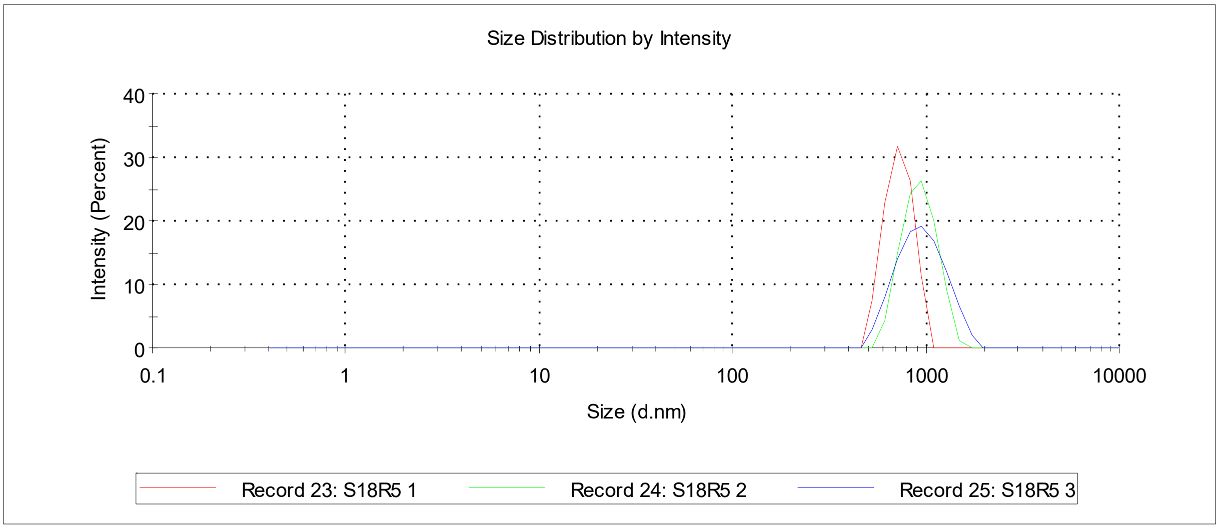

Figure S5: Size distribution of run 5

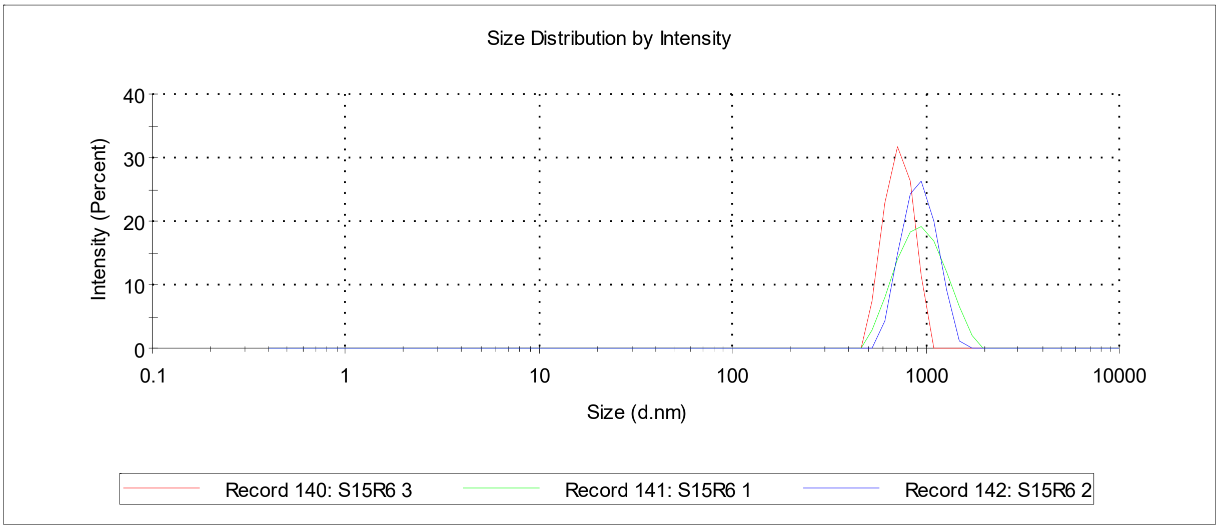

Figure S6: Size distribution of run 6

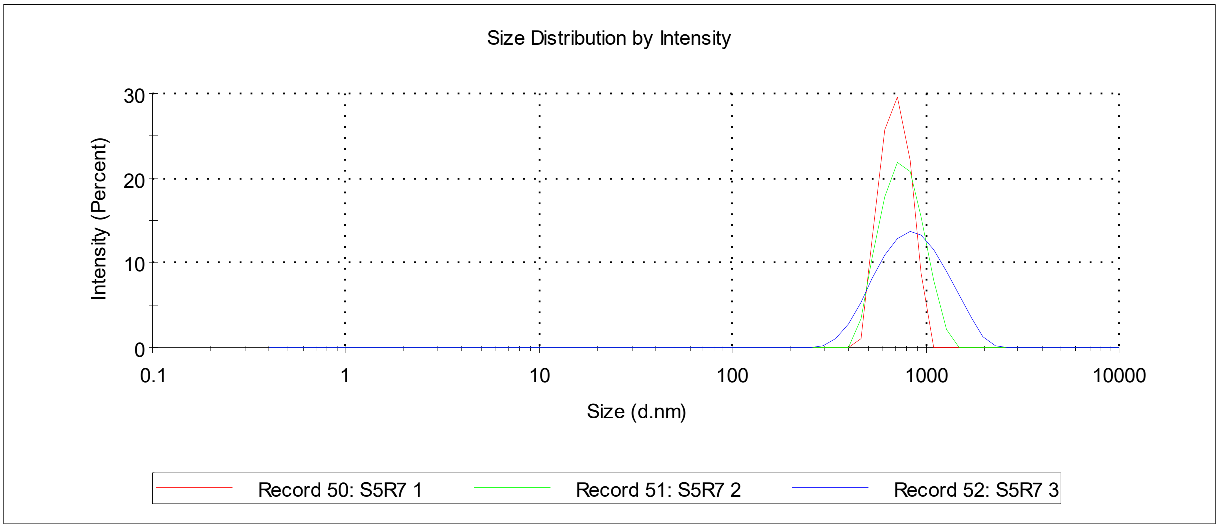

Figure S7: Size distribution of run 7

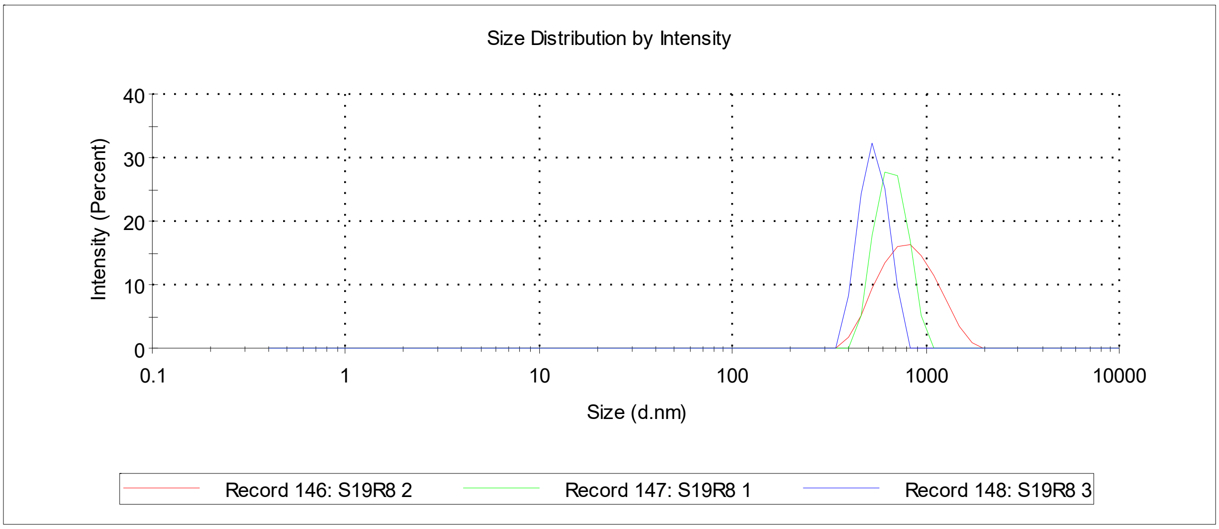

Figure S8: Size distribution of run 8

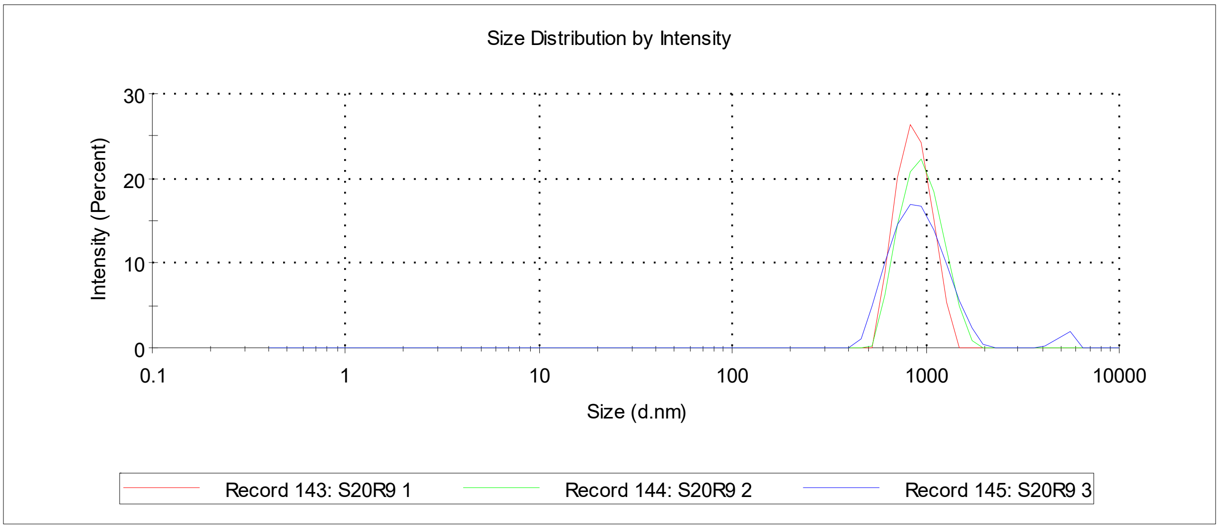

Figure S9: Size distribution of run 9

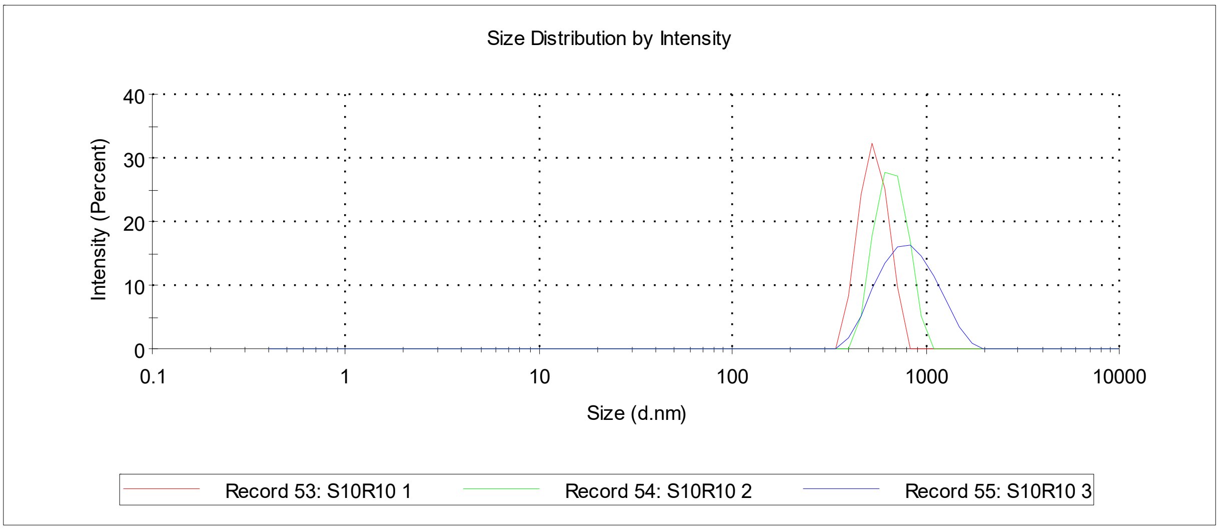

Figure S10: Size distribution of run 10

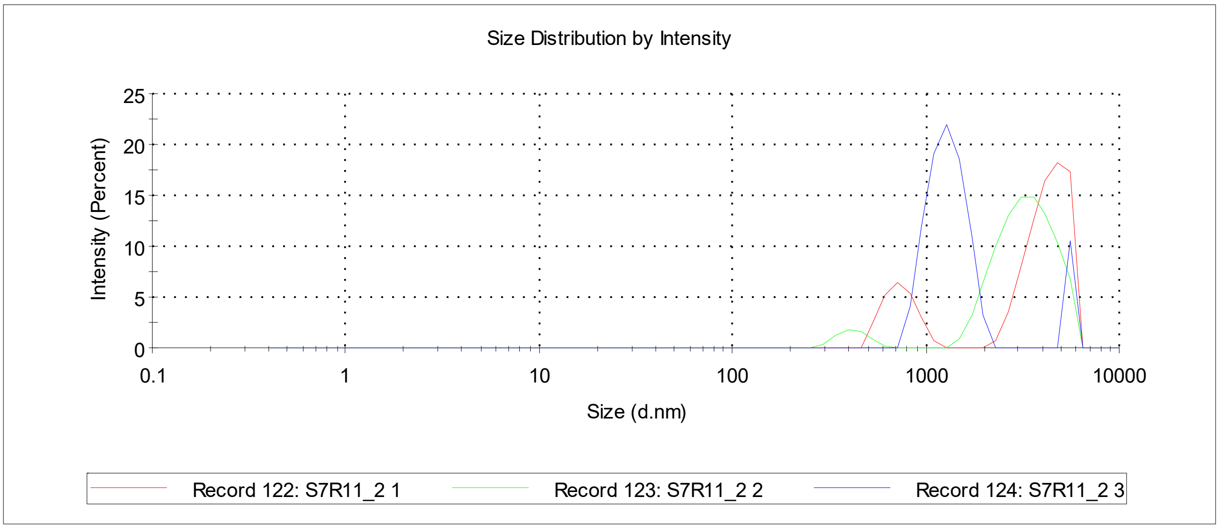

Figure S11: Size distribution of run 11

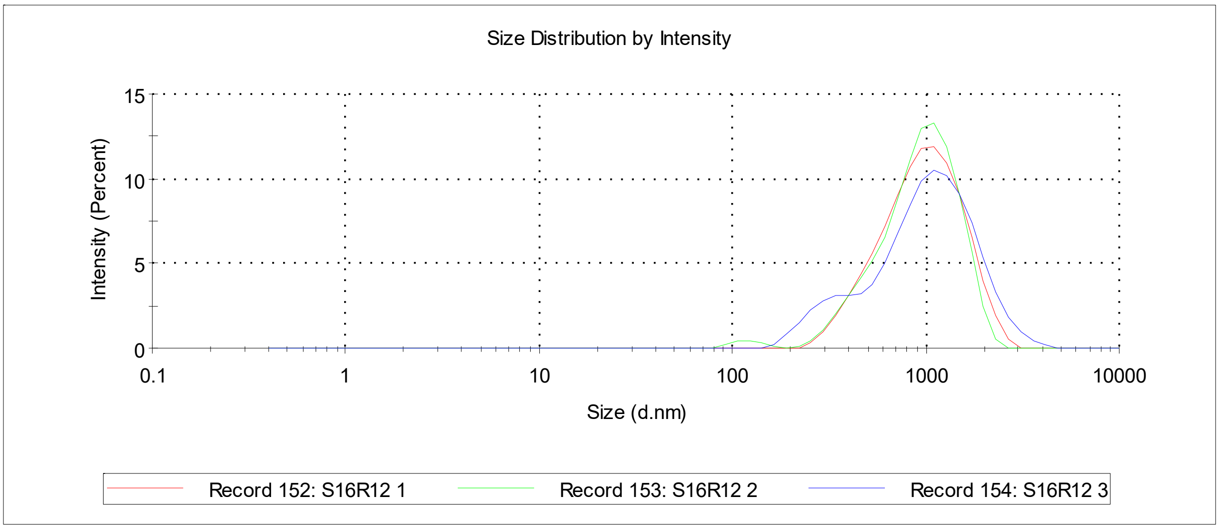

Figure S12: Size distribution of run 12

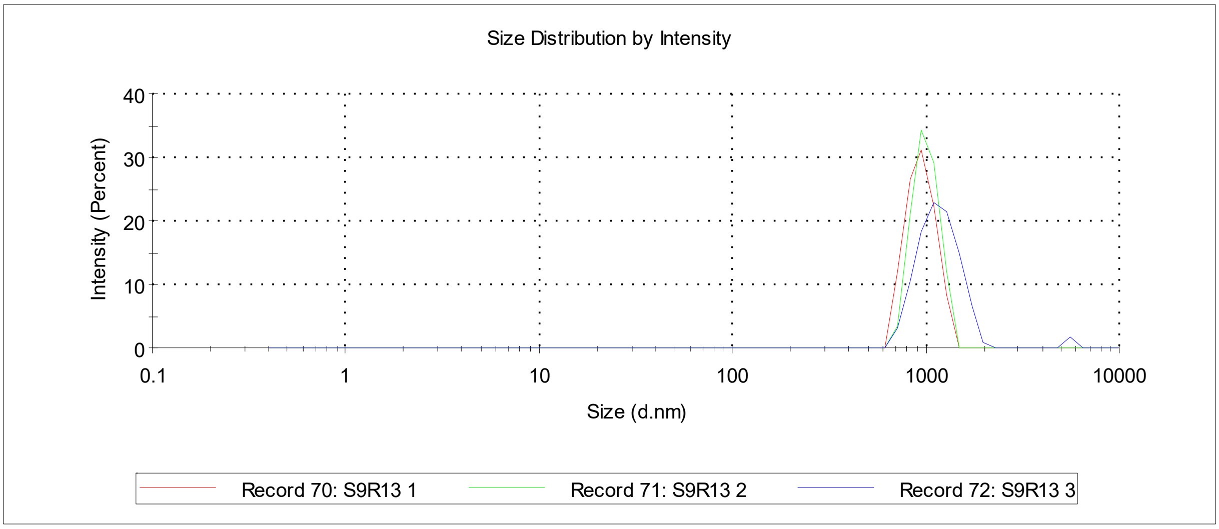

Figure S13: Size distribution of run 13

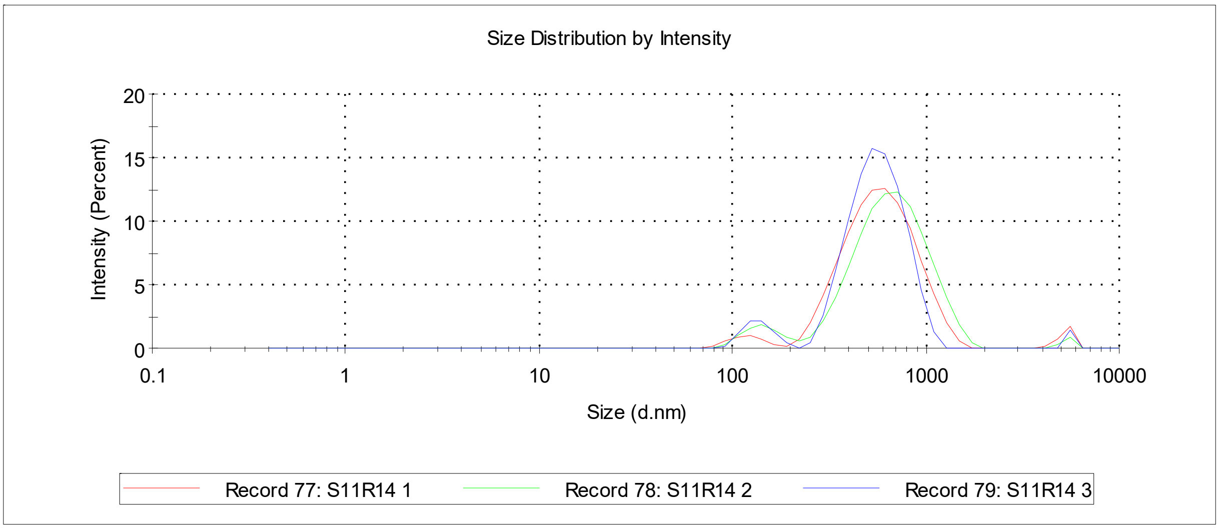

Figure S14: Size distribution of run 14

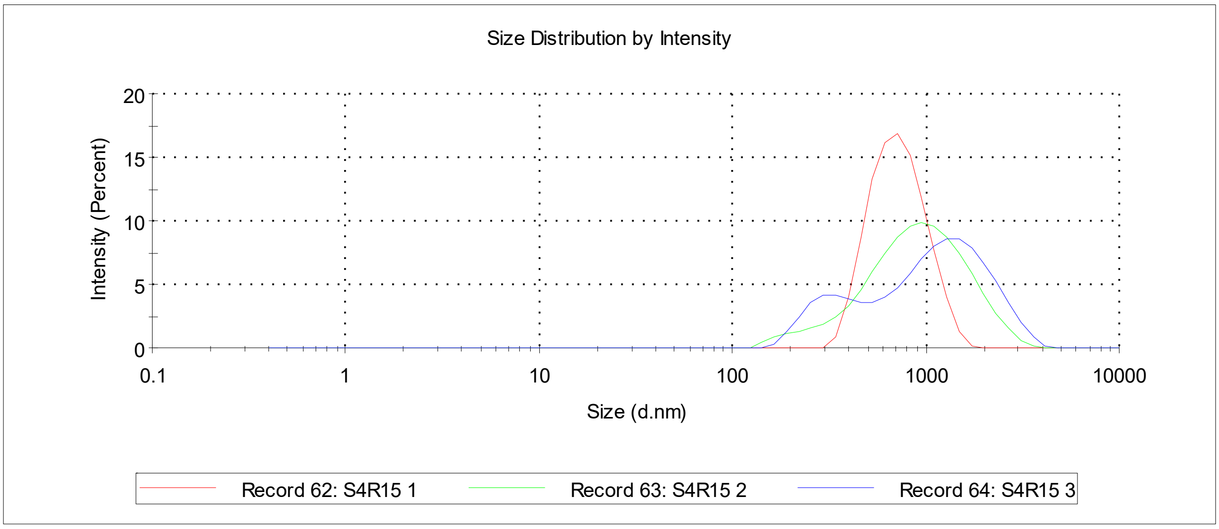

Figure S15: Size distribution of run 15

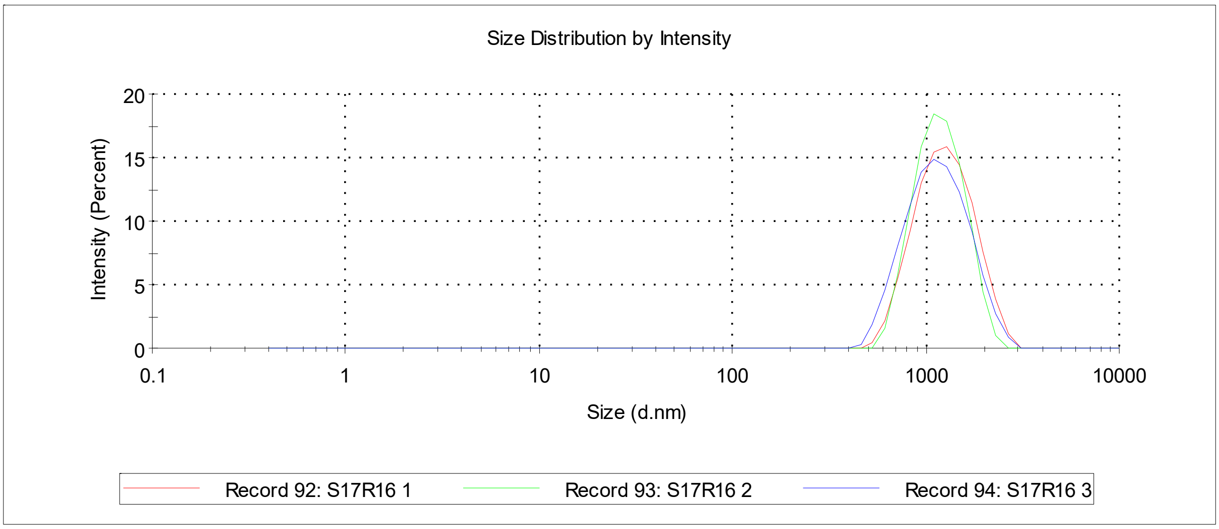

Figure S16: Size distribution of run 16

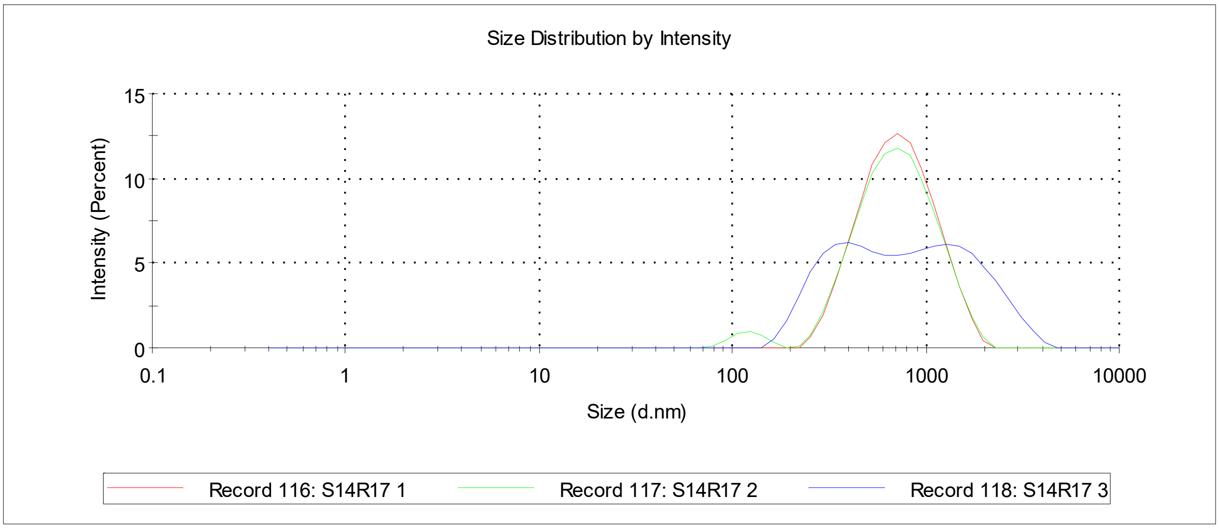

Figure S17: Size distribution of run 17

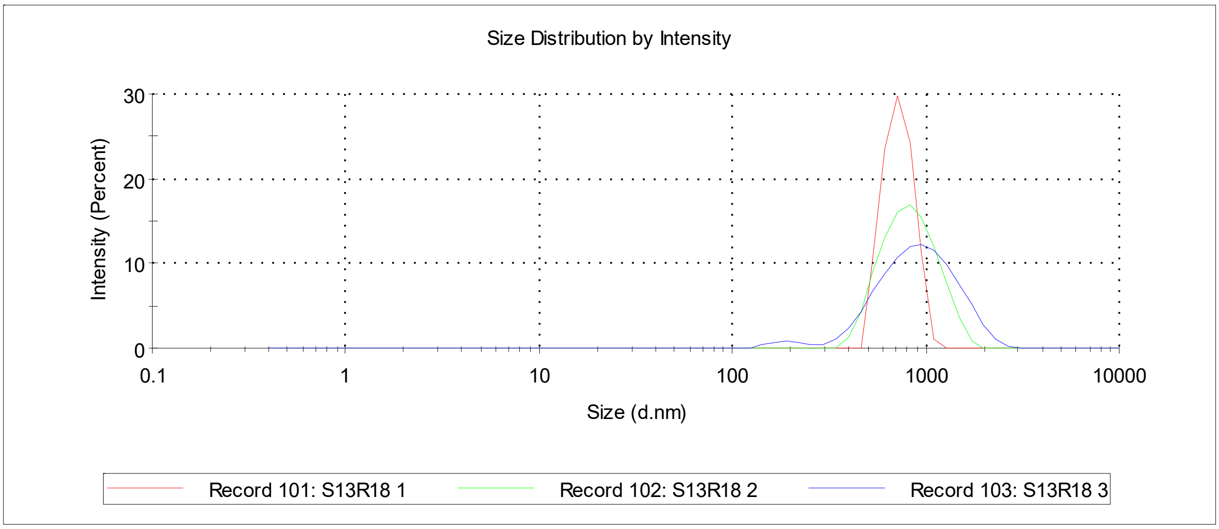

Figure S18: Size distribution of run 18

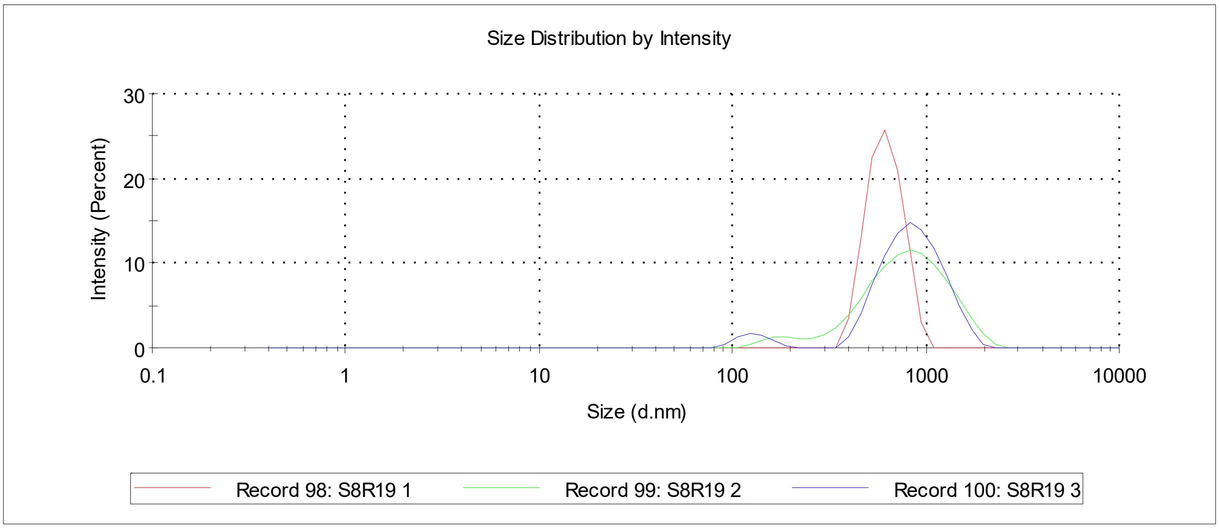

Figure S19: Size distribution of run 19

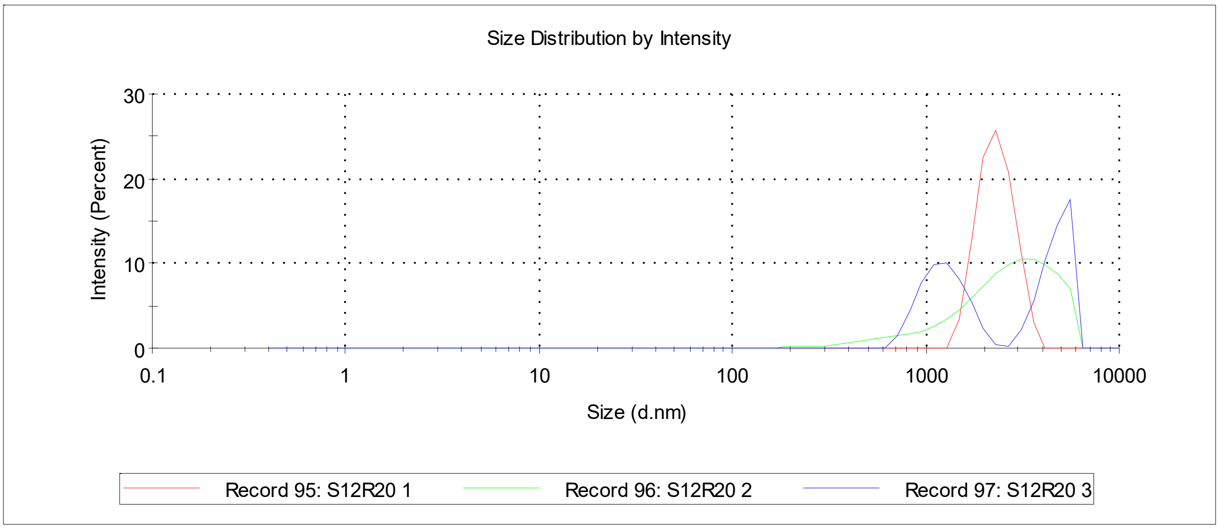

Figure S20: Size distribution of run 20
